# Supplementary figures and images for: EnsemV3X: a novel ensembled deep learning architecture for multi-label scene classification (part 2 of 2)
Source: PeerJ Comput Sci. 2021 May 25;7:e557. doi: 10.7717/peerj-cs.557 (PMC8176534; doi:10.7717/peerj-cs.557)

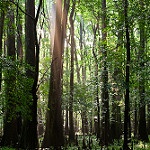

Supplement: Supplemental Information 2 [file peerj-cs-07-557-s002.zip › images/test/seg_test/forest/20447.jpg]

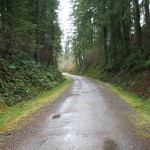

Supplement: Supplemental Information 2 [file peerj-cs-07-557-s002.zip › images/test/seg_test/forest/20448.jpg]

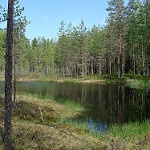

Supplement: Supplemental Information 2 [file peerj-cs-07-557-s002.zip › images/test/seg_test/forest/20450.jpg]

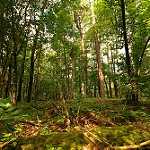

Supplement: Supplemental Information 2 [file peerj-cs-07-557-s002.zip › images/test/seg_test/forest/20485.jpg]

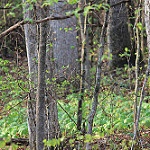

Supplement: Supplemental Information 2 [file peerj-cs-07-557-s002.zip › images/test/seg_test/forest/20502.jpg]

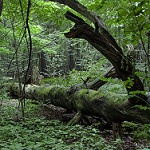

Supplement: Supplemental Information 2 [file peerj-cs-07-557-s002.zip › images/test/seg_test/forest/20510.jpg]

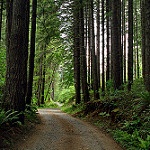

Supplement: Supplemental Information 2 [file peerj-cs-07-557-s002.zip › images/test/seg_test/forest/20533.jpg]

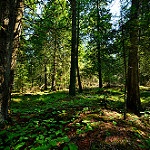

Supplement: Supplemental Information 2 [file peerj-cs-07-557-s002.zip › images/test/seg_test/forest/20544.jpg]

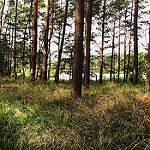

Supplement: Supplemental Information 2 [file peerj-cs-07-557-s002.zip › images/test/seg_test/forest/20556.jpg]

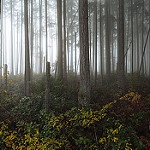

Supplement: Supplemental Information 2 [file peerj-cs-07-557-s002.zip › images/test/seg_test/forest/20563.jpg]

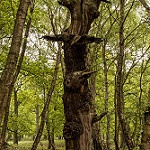

Supplement: Supplemental Information 2 [file peerj-cs-07-557-s002.zip › images/test/seg_test/forest/20568.jpg]

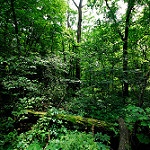

Supplement: Supplemental Information 2 [file peerj-cs-07-557-s002.zip › images/test/seg_test/forest/20582.jpg]

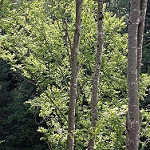

Supplement: Supplemental Information 2 [file peerj-cs-07-557-s002.zip › images/test/seg_test/forest/20596.jpg]

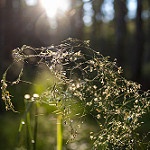

Supplement: Supplemental Information 2 [file peerj-cs-07-557-s002.zip › images/test/seg_test/forest/20605.jpg]

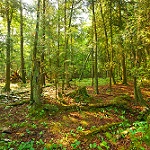

Supplement: Supplemental Information 2 [file peerj-cs-07-557-s002.zip › images/test/seg_test/forest/20619.jpg]

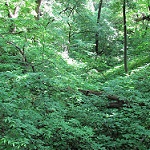

Supplement: Supplemental Information 2 [file peerj-cs-07-557-s002.zip › images/test/seg_test/forest/20620.jpg]

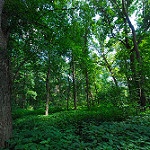

Supplement: Supplemental Information 2 [file peerj-cs-07-557-s002.zip › images/test/seg_test/forest/20638.jpg]

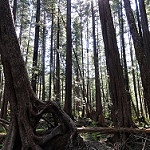

Supplement: Supplemental Information 2 [file peerj-cs-07-557-s002.zip › images/test/seg_test/forest/20650.jpg]

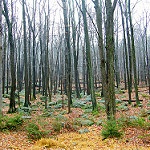

Supplement: Supplemental Information 2 [file peerj-cs-07-557-s002.zip › images/test/seg_test/forest/20660.jpg]

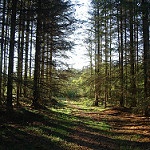

Supplement: Supplemental Information 2 [file peerj-cs-07-557-s002.zip › images/test/seg_test/forest/20674.jpg]

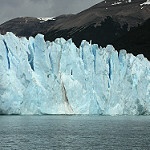

Supplement: Supplemental Information 2 [file peerj-cs-07-557-s002.zip › images/test/seg_test/glacier/20059.jpg]

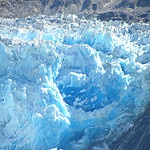

Supplement: Supplemental Information 2 [file peerj-cs-07-557-s002.zip › images/test/seg_test/glacier/20087.jpg]

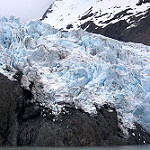

Supplement: Supplemental Information 2 [file peerj-cs-07-557-s002.zip › images/test/seg_test/glacier/20092.jpg]

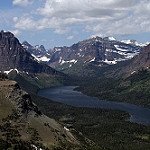

Supplement: Supplemental Information 2 [file peerj-cs-07-557-s002.zip › images/test/seg_test/glacier/20109.jpg]

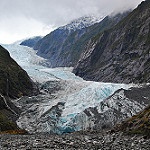

Supplement: Supplemental Information 2 [file peerj-cs-07-557-s002.zip › images/test/seg_test/glacier/20111.jpg]

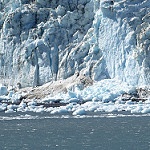

Supplement: Supplemental Information 2 [file peerj-cs-07-557-s002.zip › images/test/seg_test/glacier/20142.jpg]

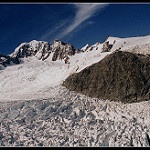

Supplement: Supplemental Information 2 [file peerj-cs-07-557-s002.zip › images/test/seg_test/glacier/20164.jpg]

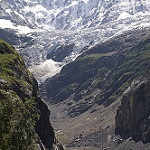

Supplement: Supplemental Information 2 [file peerj-cs-07-557-s002.zip › images/test/seg_test/glacier/20179.jpg]

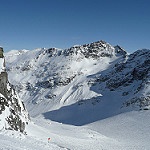

Supplement: Supplemental Information 2 [file peerj-cs-07-557-s002.zip › images/test/seg_test/glacier/20187.jpg]

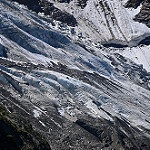

Supplement: Supplemental Information 2 [file peerj-cs-07-557-s002.zip › images/test/seg_test/glacier/20195.jpg]

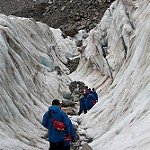

Supplement: Supplemental Information 2 [file peerj-cs-07-557-s002.zip › images/test/seg_test/glacier/20198.jpg]

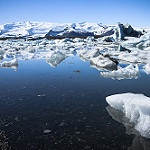

Supplement: Supplemental Information 2 [file peerj-cs-07-557-s002.zip › images/test/seg_test/glacier/20202.jpg]

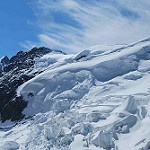

Supplement: Supplemental Information 2 [file peerj-cs-07-557-s002.zip › images/test/seg_test/glacier/20204.jpg]

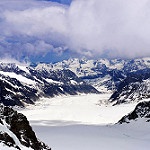

Supplement: Supplemental Information 2 [file peerj-cs-07-557-s002.zip › images/test/seg_test/glacier/20210.jpg]

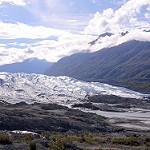

Supplement: Supplemental Information 2 [file peerj-cs-07-557-s002.zip › images/test/seg_test/glacier/20211.jpg]

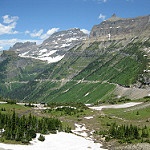

Supplement: Supplemental Information 2 [file peerj-cs-07-557-s002.zip › images/test/seg_test/glacier/20227.jpg]

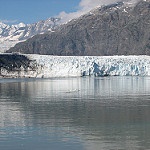

Supplement: Supplemental Information 2 [file peerj-cs-07-557-s002.zip › images/test/seg_test/glacier/20230.jpg]

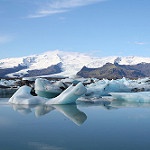

Supplement: Supplemental Information 2 [file peerj-cs-07-557-s002.zip › images/test/seg_test/glacier/20243.jpg]

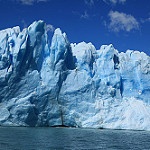

Supplement: Supplemental Information 2 [file peerj-cs-07-557-s002.zip › images/test/seg_test/glacier/20249.jpg]

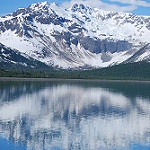

Supplement: Supplemental Information 2 [file peerj-cs-07-557-s002.zip › images/test/seg_test/glacier/20253.jpg]

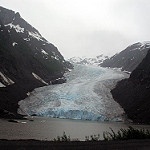

Supplement: Supplemental Information 2 [file peerj-cs-07-557-s002.zip › images/test/seg_test/glacier/20272.jpg]

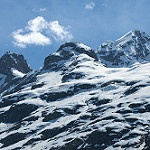

Supplement: Supplemental Information 2 [file peerj-cs-07-557-s002.zip › images/test/seg_test/glacier/20275.jpg]

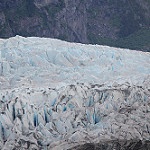

Supplement: Supplemental Information 2 [file peerj-cs-07-557-s002.zip › images/test/seg_test/glacier/20287.jpg]

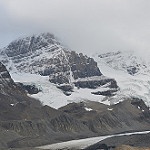

Supplement: Supplemental Information 2 [file peerj-cs-07-557-s002.zip › images/test/seg_test/glacier/20292.jpg]

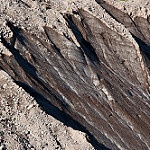

Supplement: Supplemental Information 2 [file peerj-cs-07-557-s002.zip › images/test/seg_test/glacier/20303.jpg]

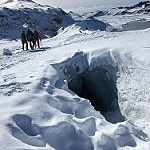

Supplement: Supplemental Information 2 [file peerj-cs-07-557-s002.zip › images/test/seg_test/glacier/20307.jpg]

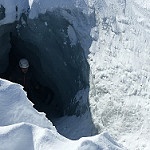

Supplement: Supplemental Information 2 [file peerj-cs-07-557-s002.zip › images/test/seg_test/glacier/20310.jpg]

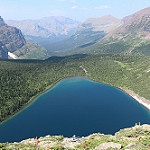

Supplement: Supplemental Information 2 [file peerj-cs-07-557-s002.zip › images/test/seg_test/glacier/20317.jpg]

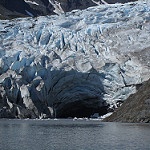

Supplement: Supplemental Information 2 [file peerj-cs-07-557-s002.zip › images/test/seg_test/glacier/20326.jpg]

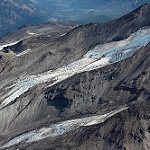

Supplement: Supplemental Information 2 [file peerj-cs-07-557-s002.zip › images/test/seg_test/glacier/20329.jpg]

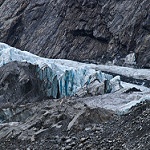

Supplement: Supplemental Information 2 [file peerj-cs-07-557-s002.zip › images/test/seg_test/glacier/20345.jpg]

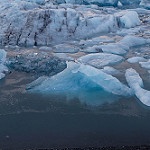

Supplement: Supplemental Information 2 [file peerj-cs-07-557-s002.zip › images/test/seg_test/glacier/20352.jpg]

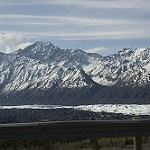

Supplement: Supplemental Information 2 [file peerj-cs-07-557-s002.zip › images/test/seg_test/glacier/20355.jpg]

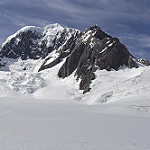

Supplement: Supplemental Information 2 [file peerj-cs-07-557-s002.zip › images/test/seg_test/glacier/20362.jpg]

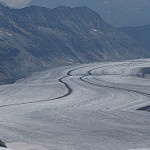

Supplement: Supplemental Information 2 [file peerj-cs-07-557-s002.zip › images/test/seg_test/glacier/20372.jpg]

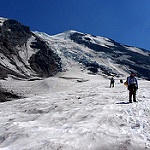

Supplement: Supplemental Information 2 [file peerj-cs-07-557-s002.zip › images/test/seg_test/glacier/20373.jpg]

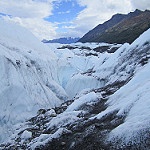

Supplement: Supplemental Information 2 [file peerj-cs-07-557-s002.zip › images/test/seg_test/glacier/20380.jpg]

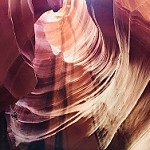

Supplement: Supplemental Information 2 [file peerj-cs-07-557-s002.zip › images/test/seg_test/glacier/20383.jpg]

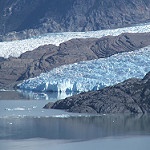

Supplement: Supplemental Information 2 [file peerj-cs-07-557-s002.zip › images/test/seg_test/glacier/20386.jpg]

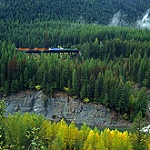

Supplement: Supplemental Information 2 [file peerj-cs-07-557-s002.zip › images/test/seg_test/glacier/20387.jpg]

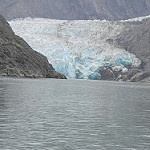

Supplement: Supplemental Information 2 [file peerj-cs-07-557-s002.zip › images/test/seg_test/glacier/20406.jpg]

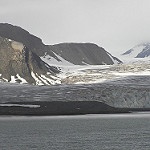

Supplement: Supplemental Information 2 [file peerj-cs-07-557-s002.zip › images/test/seg_test/glacier/20409.jpg]

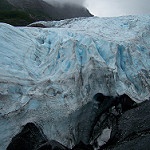

Supplement: Supplemental Information 2 [file peerj-cs-07-557-s002.zip › images/test/seg_test/glacier/20413.jpg]

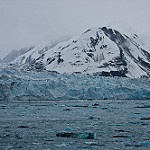

Supplement: Supplemental Information 2 [file peerj-cs-07-557-s002.zip › images/test/seg_test/glacier/20414.jpg]

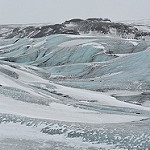

Supplement: Supplemental Information 2 [file peerj-cs-07-557-s002.zip › images/test/seg_test/glacier/20418.jpg]

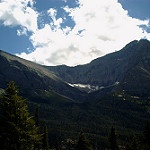

Supplement: Supplemental Information 2 [file peerj-cs-07-557-s002.zip › images/test/seg_test/glacier/20421.jpg]

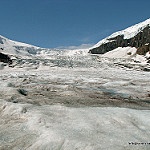

Supplement: Supplemental Information 2 [file peerj-cs-07-557-s002.zip › images/test/seg_test/glacier/20423.jpg]

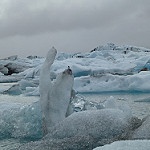

Supplement: Supplemental Information 2 [file peerj-cs-07-557-s002.zip › images/test/seg_test/glacier/20433.jpg]

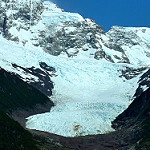

Supplement: Supplemental Information 2 [file peerj-cs-07-557-s002.zip › images/test/seg_test/glacier/20446.jpg]

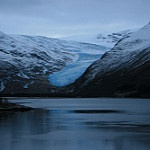

Supplement: Supplemental Information 2 [file peerj-cs-07-557-s002.zip › images/test/seg_test/glacier/20457.jpg]

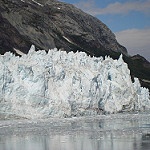

Supplement: Supplemental Information 2 [file peerj-cs-07-557-s002.zip › images/test/seg_test/glacier/20458.jpg]

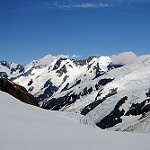

Supplement: Supplemental Information 2 [file peerj-cs-07-557-s002.zip › images/test/seg_test/glacier/20467.jpg]

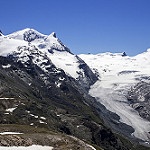

Supplement: Supplemental Information 2 [file peerj-cs-07-557-s002.zip › images/test/seg_test/glacier/20475.jpg]

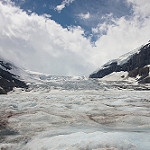

Supplement: Supplemental Information 2 [file peerj-cs-07-557-s002.zip › images/test/seg_test/glacier/20477.jpg]

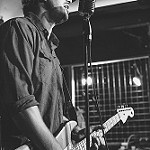

Supplement: Supplemental Information 2 [file peerj-cs-07-557-s002.zip › images/test/seg_test/glacier/20480.jpg]

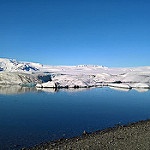

Supplement: Supplemental Information 2 [file peerj-cs-07-557-s002.zip › images/test/seg_test/glacier/20482.jpg]

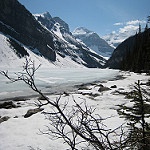

Supplement: Supplemental Information 2 [file peerj-cs-07-557-s002.zip › images/test/seg_test/glacier/20486.jpg]

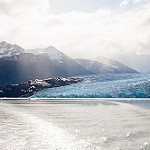

Supplement: Supplemental Information 2 [file peerj-cs-07-557-s002.zip › images/test/seg_test/glacier/20489.jpg]

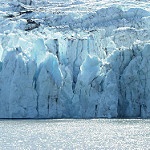

Supplement: Supplemental Information 2 [file peerj-cs-07-557-s002.zip › images/test/seg_test/glacier/20490.jpg]

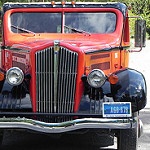

Supplement: Supplemental Information 2 [file peerj-cs-07-557-s002.zip › images/test/seg_test/glacier/20491.jpg]

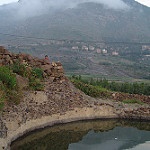

Supplement: Supplemental Information 2 [file peerj-cs-07-557-s002.zip › images/test/seg_test/mountain/20058.jpg]

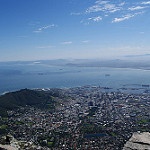

Supplement: Supplemental Information 2 [file peerj-cs-07-557-s002.zip › images/test/seg_test/mountain/20068.jpg]

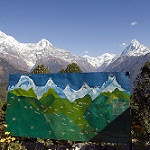

Supplement: Supplemental Information 2 [file peerj-cs-07-557-s002.zip › images/test/seg_test/mountain/20071.jpg]

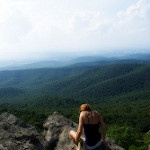

Supplement: Supplemental Information 2 [file peerj-cs-07-557-s002.zip › images/test/seg_test/mountain/20085.jpg]

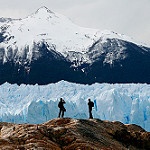

Supplement: Supplemental Information 2 [file peerj-cs-07-557-s002.zip › images/test/seg_test/mountain/20093.jpg]

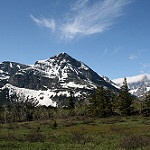

Supplement: Supplemental Information 2 [file peerj-cs-07-557-s002.zip › images/test/seg_test/mountain/20107.jpg]

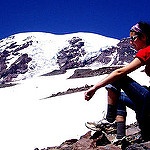

Supplement: Supplemental Information 2 [file peerj-cs-07-557-s002.zip › images/test/seg_test/mountain/20116.jpg]

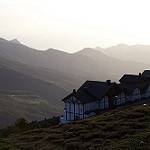

Supplement: Supplemental Information 2 [file peerj-cs-07-557-s002.zip › images/test/seg_test/mountain/20120.jpg]

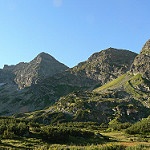

Supplement: Supplemental Information 2 [file peerj-cs-07-557-s002.zip › images/test/seg_test/mountain/20123.jpg]

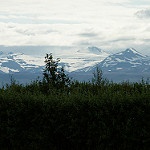

Supplement: Supplemental Information 2 [file peerj-cs-07-557-s002.zip › images/test/seg_test/mountain/20129.jpg]

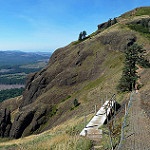

Supplement: Supplemental Information 2 [file peerj-cs-07-557-s002.zip › images/test/seg_test/mountain/20133.jpg]

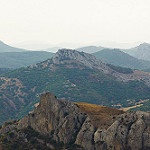

Supplement: Supplemental Information 2 [file peerj-cs-07-557-s002.zip › images/test/seg_test/mountain/20144.jpg]

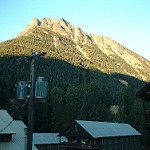

Supplement: Supplemental Information 2 [file peerj-cs-07-557-s002.zip › images/test/seg_test/mountain/20153.jpg]

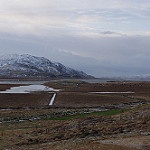

Supplement: Supplemental Information 2 [file peerj-cs-07-557-s002.zip › images/test/seg_test/mountain/20154.jpg]

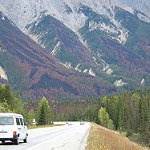

Supplement: Supplemental Information 2 [file peerj-cs-07-557-s002.zip › images/test/seg_test/mountain/20157.jpg]

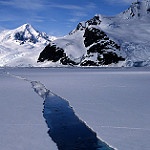

Supplement: Supplemental Information 2 [file peerj-cs-07-557-s002.zip › images/test/seg_test/mountain/20160.jpg]

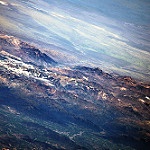

Supplement: Supplemental Information 2 [file peerj-cs-07-557-s002.zip › images/test/seg_test/mountain/20174.jpg]

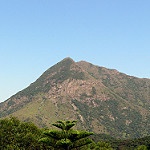

Supplement: Supplemental Information 2 [file peerj-cs-07-557-s002.zip › images/test/seg_test/mountain/20176.jpg]

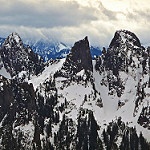

Supplement: Supplemental Information 2 [file peerj-cs-07-557-s002.zip › images/test/seg_test/mountain/20181.jpg]

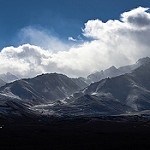

Supplement: Supplemental Information 2 [file peerj-cs-07-557-s002.zip › images/test/seg_test/mountain/20189.jpg]
