# Supplementary figures and images for: EnsemV3X: a novel ensembled deep learning architecture for multi-label scene classification (part 1 of 2)
Source: PeerJ Comput Sci. 2021 May 25;7:e557. doi: 10.7717/peerj-cs.557 (PMC8176534; doi:10.7717/peerj-cs.557)

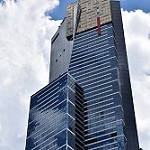

Supplement: Supplemental Information 2 [file peerj-cs-07-557-s002.zip › images/test/seg_test/buildings/20057.jpg]

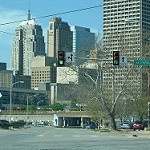

Supplement: Supplemental Information 2 [file peerj-cs-07-557-s002.zip › images/test/seg_test/buildings/20060.jpg]

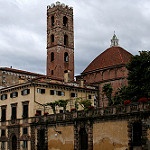

Supplement: Supplemental Information 2 [file peerj-cs-07-557-s002.zip › images/test/seg_test/buildings/20061.jpg]

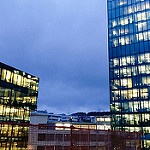

Supplement: Supplemental Information 2 [file peerj-cs-07-557-s002.zip › images/test/seg_test/buildings/20064.jpg]

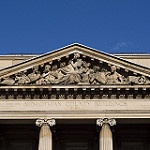

Supplement: Supplemental Information 2 [file peerj-cs-07-557-s002.zip › images/test/seg_test/buildings/20073.jpg]

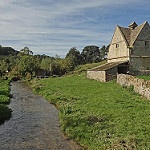

Supplement: Supplemental Information 2 [file peerj-cs-07-557-s002.zip › images/test/seg_test/buildings/20074.jpg]

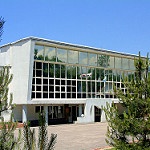

Supplement: Supplemental Information 2 [file peerj-cs-07-557-s002.zip › images/test/seg_test/buildings/20078.jpg]

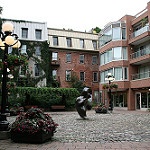

Supplement: Supplemental Information 2 [file peerj-cs-07-557-s002.zip › images/test/seg_test/buildings/20083.jpg]

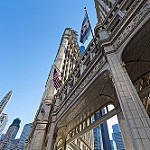

Supplement: Supplemental Information 2 [file peerj-cs-07-557-s002.zip › images/test/seg_test/buildings/20094.jpg]

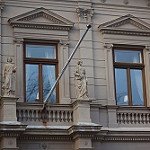

Supplement: Supplemental Information 2 [file peerj-cs-07-557-s002.zip › images/test/seg_test/buildings/20096.jpg]

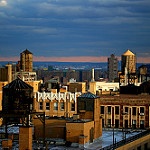

Supplement: Supplemental Information 2 [file peerj-cs-07-557-s002.zip › images/test/seg_test/buildings/20113.jpg]

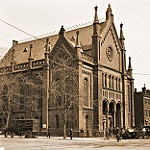

Supplement: Supplemental Information 2 [file peerj-cs-07-557-s002.zip › images/test/seg_test/buildings/20131.jpg]

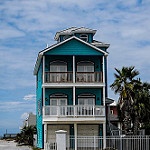

Supplement: Supplemental Information 2 [file peerj-cs-07-557-s002.zip › images/test/seg_test/buildings/20140.jpg]

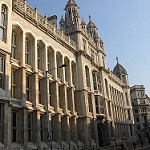

Supplement: Supplemental Information 2 [file peerj-cs-07-557-s002.zip › images/test/seg_test/buildings/20177.jpg]

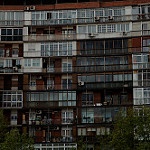

Supplement: Supplemental Information 2 [file peerj-cs-07-557-s002.zip › images/test/seg_test/buildings/20186.jpg]

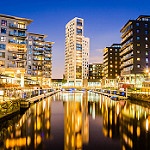

Supplement: Supplemental Information 2 [file peerj-cs-07-557-s002.zip › images/test/seg_test/buildings/20206.jpg]

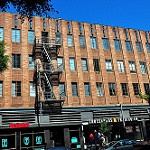

Supplement: Supplemental Information 2 [file peerj-cs-07-557-s002.zip › images/test/seg_test/buildings/20207.jpg]

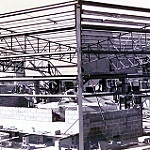

Supplement: Supplemental Information 2 [file peerj-cs-07-557-s002.zip › images/test/seg_test/buildings/20218.jpg]

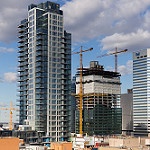

Supplement: Supplemental Information 2 [file peerj-cs-07-557-s002.zip › images/test/seg_test/buildings/20228.jpg]

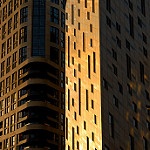

Supplement: Supplemental Information 2 [file peerj-cs-07-557-s002.zip › images/test/seg_test/buildings/20231.jpg]

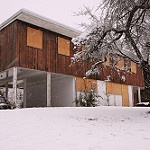

Supplement: Supplemental Information 2 [file peerj-cs-07-557-s002.zip › images/test/seg_test/buildings/20241.jpg]

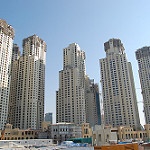

Supplement: Supplemental Information 2 [file peerj-cs-07-557-s002.zip › images/test/seg_test/buildings/20245.jpg]

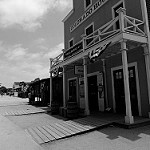

Supplement: Supplemental Information 2 [file peerj-cs-07-557-s002.zip › images/test/seg_test/buildings/20246.jpg]

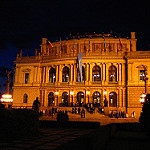

Supplement: Supplemental Information 2 [file peerj-cs-07-557-s002.zip › images/test/seg_test/buildings/20250.jpg]

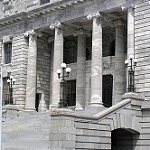

Supplement: Supplemental Information 2 [file peerj-cs-07-557-s002.zip › images/test/seg_test/buildings/20268.jpg]

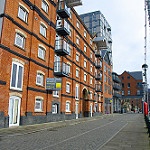

Supplement: Supplemental Information 2 [file peerj-cs-07-557-s002.zip › images/test/seg_test/buildings/20294.jpg]

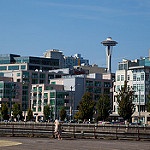

Supplement: Supplemental Information 2 [file peerj-cs-07-557-s002.zip › images/test/seg_test/buildings/20309.jpg]

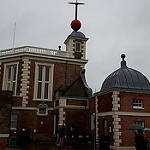

Supplement: Supplemental Information 2 [file peerj-cs-07-557-s002.zip › images/test/seg_test/buildings/20344.jpg]

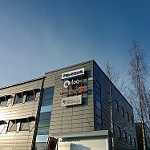

Supplement: Supplemental Information 2 [file peerj-cs-07-557-s002.zip › images/test/seg_test/buildings/20350.jpg]

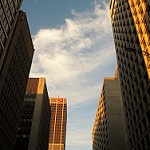

Supplement: Supplemental Information 2 [file peerj-cs-07-557-s002.zip › images/test/seg_test/buildings/20366.jpg]

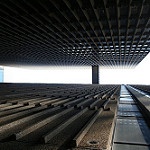

Supplement: Supplemental Information 2 [file peerj-cs-07-557-s002.zip › images/test/seg_test/buildings/20374.jpg]

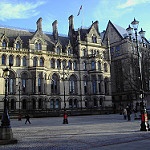

Supplement: Supplemental Information 2 [file peerj-cs-07-557-s002.zip › images/test/seg_test/buildings/20390.jpg]

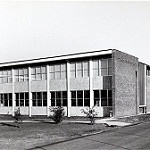

Supplement: Supplemental Information 2 [file peerj-cs-07-557-s002.zip › images/test/seg_test/buildings/20394.jpg]

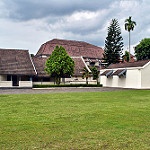

Supplement: Supplemental Information 2 [file peerj-cs-07-557-s002.zip › images/test/seg_test/buildings/20424.jpg]

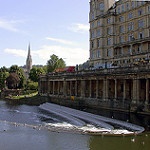

Supplement: Supplemental Information 2 [file peerj-cs-07-557-s002.zip › images/test/seg_test/buildings/20425.jpg]

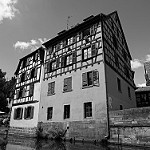

Supplement: Supplemental Information 2 [file peerj-cs-07-557-s002.zip › images/test/seg_test/buildings/20430.jpg]

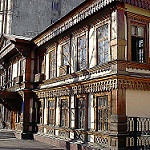

Supplement: Supplemental Information 2 [file peerj-cs-07-557-s002.zip › images/test/seg_test/buildings/20431.jpg]

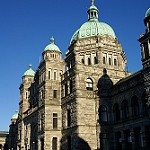

Supplement: Supplemental Information 2 [file peerj-cs-07-557-s002.zip › images/test/seg_test/buildings/20436.jpg]

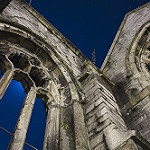

Supplement: Supplemental Information 2 [file peerj-cs-07-557-s002.zip › images/test/seg_test/buildings/20441.jpg]

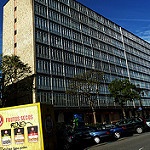

Supplement: Supplemental Information 2 [file peerj-cs-07-557-s002.zip › images/test/seg_test/buildings/20449.jpg]

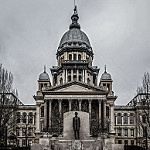

Supplement: Supplemental Information 2 [file peerj-cs-07-557-s002.zip › images/test/seg_test/buildings/20460.jpg]

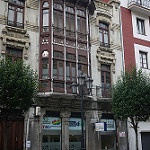

Supplement: Supplemental Information 2 [file peerj-cs-07-557-s002.zip › images/test/seg_test/buildings/20465.jpg]

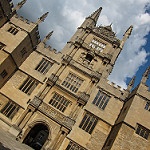

Supplement: Supplemental Information 2 [file peerj-cs-07-557-s002.zip › images/test/seg_test/buildings/20504.jpg]

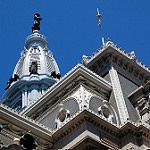

Supplement: Supplemental Information 2 [file peerj-cs-07-557-s002.zip › images/test/seg_test/buildings/20515.jpg]

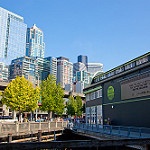

Supplement: Supplemental Information 2 [file peerj-cs-07-557-s002.zip › images/test/seg_test/buildings/20524.jpg]

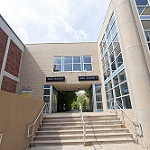

Supplement: Supplemental Information 2 [file peerj-cs-07-557-s002.zip › images/test/seg_test/buildings/20536.jpg]

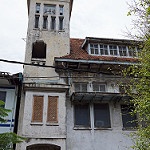

Supplement: Supplemental Information 2 [file peerj-cs-07-557-s002.zip › images/test/seg_test/buildings/20546.jpg]

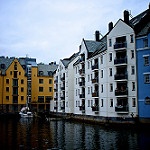

Supplement: Supplemental Information 2 [file peerj-cs-07-557-s002.zip › images/test/seg_test/buildings/20549.jpg]

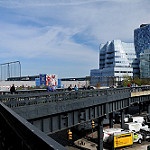

Supplement: Supplemental Information 2 [file peerj-cs-07-557-s002.zip › images/test/seg_test/buildings/20550.jpg]

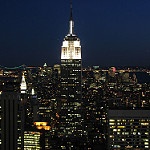

Supplement: Supplemental Information 2 [file peerj-cs-07-557-s002.zip › images/test/seg_test/buildings/20553.jpg]

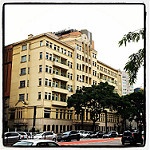

Supplement: Supplemental Information 2 [file peerj-cs-07-557-s002.zip › images/test/seg_test/buildings/20555.jpg]

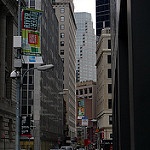

Supplement: Supplemental Information 2 [file peerj-cs-07-557-s002.zip › images/test/seg_test/buildings/20574.jpg]

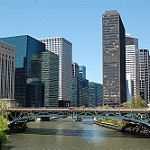

Supplement: Supplemental Information 2 [file peerj-cs-07-557-s002.zip › images/test/seg_test/buildings/20580.jpg]

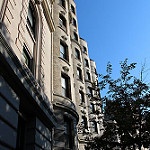

Supplement: Supplemental Information 2 [file peerj-cs-07-557-s002.zip › images/test/seg_test/buildings/20581.jpg]

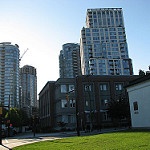

Supplement: Supplemental Information 2 [file peerj-cs-07-557-s002.zip › images/test/seg_test/buildings/20587.jpg]

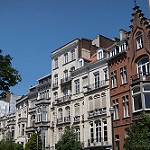

Supplement: Supplemental Information 2 [file peerj-cs-07-557-s002.zip › images/test/seg_test/buildings/20597.jpg]

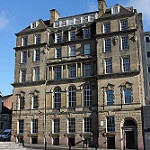

Supplement: Supplemental Information 2 [file peerj-cs-07-557-s002.zip › images/test/seg_test/buildings/20601.jpg]

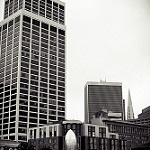

Supplement: Supplemental Information 2 [file peerj-cs-07-557-s002.zip › images/test/seg_test/buildings/20606.jpg]

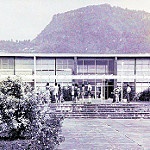

Supplement: Supplemental Information 2 [file peerj-cs-07-557-s002.zip › images/test/seg_test/buildings/20611.jpg]

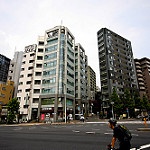

Supplement: Supplemental Information 2 [file peerj-cs-07-557-s002.zip › images/test/seg_test/buildings/20628.jpg]

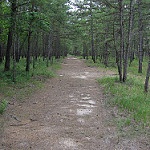

Supplement: Supplemental Information 2 [file peerj-cs-07-557-s002.zip › images/test/seg_test/forest/20056.jpg]

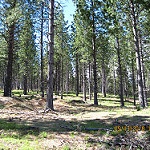

Supplement: Supplemental Information 2 [file peerj-cs-07-557-s002.zip › images/test/seg_test/forest/20062.jpg]

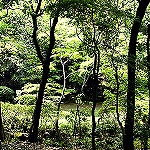

Supplement: Supplemental Information 2 [file peerj-cs-07-557-s002.zip › images/test/seg_test/forest/20082.jpg]

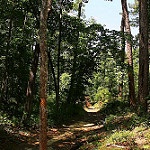

Supplement: Supplemental Information 2 [file peerj-cs-07-557-s002.zip › images/test/seg_test/forest/20089.jpg]

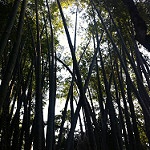

Supplement: Supplemental Information 2 [file peerj-cs-07-557-s002.zip › images/test/seg_test/forest/20091.jpg]

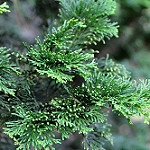

Supplement: Supplemental Information 2 [file peerj-cs-07-557-s002.zip › images/test/seg_test/forest/20095.jpg]

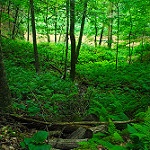

Supplement: Supplemental Information 2 [file peerj-cs-07-557-s002.zip › images/test/seg_test/forest/20098.jpg]

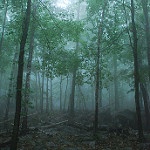

Supplement: Supplemental Information 2 [file peerj-cs-07-557-s002.zip › images/test/seg_test/forest/20100.jpg]

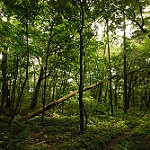

Supplement: Supplemental Information 2 [file peerj-cs-07-557-s002.zip › images/test/seg_test/forest/20108.jpg]

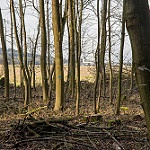

Supplement: Supplemental Information 2 [file peerj-cs-07-557-s002.zip › images/test/seg_test/forest/20117.jpg]

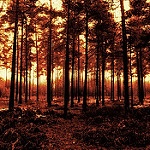

Supplement: Supplemental Information 2 [file peerj-cs-07-557-s002.zip › images/test/seg_test/forest/20134.jpg]

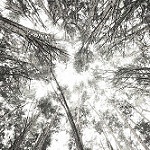

Supplement: Supplemental Information 2 [file peerj-cs-07-557-s002.zip › images/test/seg_test/forest/20136.jpg]

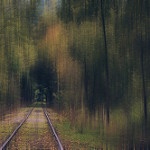

Supplement: Supplemental Information 2 [file peerj-cs-07-557-s002.zip › images/test/seg_test/forest/20147.jpg]

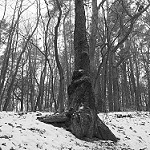

Supplement: Supplemental Information 2 [file peerj-cs-07-557-s002.zip › images/test/seg_test/forest/20150.jpg]

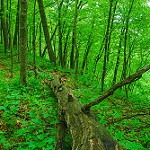

Supplement: Supplemental Information 2 [file peerj-cs-07-557-s002.zip › images/test/seg_test/forest/20151.jpg]

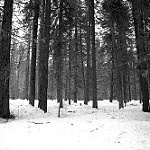

Supplement: Supplemental Information 2 [file peerj-cs-07-557-s002.zip › images/test/seg_test/forest/20159.jpg]

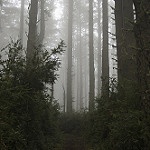

Supplement: Supplemental Information 2 [file peerj-cs-07-557-s002.zip › images/test/seg_test/forest/20166.jpg]

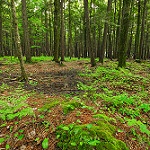

Supplement: Supplemental Information 2 [file peerj-cs-07-557-s002.zip › images/test/seg_test/forest/20173.jpg]

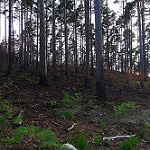

Supplement: Supplemental Information 2 [file peerj-cs-07-557-s002.zip › images/test/seg_test/forest/20175.jpg]

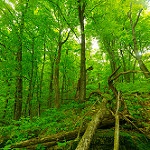

Supplement: Supplemental Information 2 [file peerj-cs-07-557-s002.zip › images/test/seg_test/forest/20225.jpg]

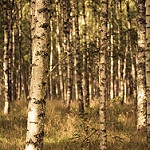

Supplement: Supplemental Information 2 [file peerj-cs-07-557-s002.zip › images/test/seg_test/forest/20229.jpg]

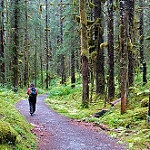

Supplement: Supplemental Information 2 [file peerj-cs-07-557-s002.zip › images/test/seg_test/forest/20242.jpg]

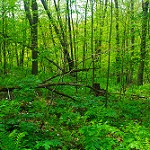

Supplement: Supplemental Information 2 [file peerj-cs-07-557-s002.zip › images/test/seg_test/forest/20252.jpg]

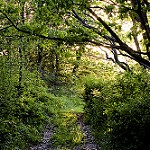

Supplement: Supplemental Information 2 [file peerj-cs-07-557-s002.zip › images/test/seg_test/forest/20260.jpg]

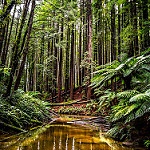

Supplement: Supplemental Information 2 [file peerj-cs-07-557-s002.zip › images/test/seg_test/forest/20261.jpg]

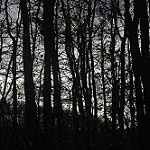

Supplement: Supplemental Information 2 [file peerj-cs-07-557-s002.zip › images/test/seg_test/forest/20274.jpg]

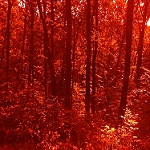

Supplement: Supplemental Information 2 [file peerj-cs-07-557-s002.zip › images/test/seg_test/forest/20288.jpg]

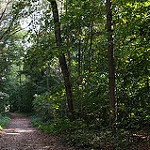

Supplement: Supplemental Information 2 [file peerj-cs-07-557-s002.zip › images/test/seg_test/forest/20299.jpg]

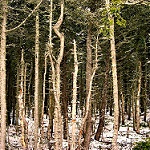

Supplement: Supplemental Information 2 [file peerj-cs-07-557-s002.zip › images/test/seg_test/forest/20306.jpg]

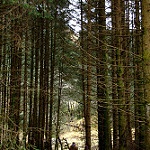

Supplement: Supplemental Information 2 [file peerj-cs-07-557-s002.zip › images/test/seg_test/forest/20311.jpg]

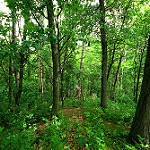

Supplement: Supplemental Information 2 [file peerj-cs-07-557-s002.zip › images/test/seg_test/forest/20315.jpg]

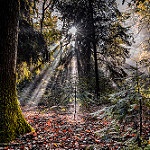

Supplement: Supplemental Information 2 [file peerj-cs-07-557-s002.zip › images/test/seg_test/forest/20319.jpg]

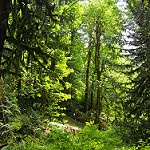

Supplement: Supplemental Information 2 [file peerj-cs-07-557-s002.zip › images/test/seg_test/forest/20322.jpg]

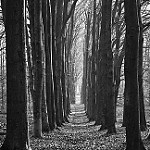

Supplement: Supplemental Information 2 [file peerj-cs-07-557-s002.zip › images/test/seg_test/forest/20328.jpg]

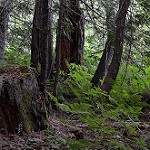

Supplement: Supplemental Information 2 [file peerj-cs-07-557-s002.zip › images/test/seg_test/forest/20330.jpg]

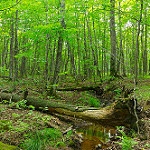

Supplement: Supplemental Information 2 [file peerj-cs-07-557-s002.zip › images/test/seg_test/forest/20384.jpg]

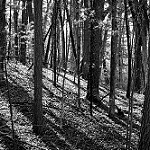

Supplement: Supplemental Information 2 [file peerj-cs-07-557-s002.zip › images/test/seg_test/forest/20400.jpg]

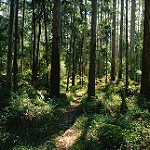

Supplement: Supplemental Information 2 [file peerj-cs-07-557-s002.zip › images/test/seg_test/forest/20411.jpg]

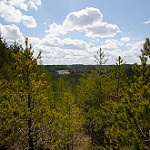

Supplement: Supplemental Information 2 [file peerj-cs-07-557-s002.zip › images/test/seg_test/forest/20416.jpg]

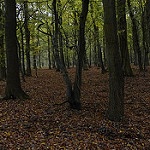

Supplement: Supplemental Information 2 [file peerj-cs-07-557-s002.zip › images/test/seg_test/forest/20439.jpg]
